# Acute treatment of migraine: quantifying the unmet need through real-world data in Italy

**DOI:** 10.1007/s10072-024-07493-w

**Published:** 2024-03-27

**Authors:** Simona Sacco, Sonia Di Ciaccio, Roberto Di Virgilio, Valeria Pegoraro, Raffaele Ornello

**Affiliations:** 1 Department of Biotechnological and Applied Clinical Sciences, University of L’Aquila, L’Aquila, Italy; 2 Medical Department, Pfizer Srl, Rome, Italy; 3 Health Economics and Outcome Research Department, Pfizer Srl, Rome, Italy; 4 RWS Department, IQVIA Solutions Italy S.R.L, Via Fabio Filzi 29, 20124 Milan, Italy

**Keywords:** Migraine, Triptans, Poor tolerability, Poor effectiveness, Contraindications, Unmet need, Real-world evidence

## Abstract

**Objective:**

This study is describing subjects with migraine interrupting or not receiving triptans for acute treatment and providing a national-level estimate of people who might benefit from different therapeutic approaches.

**Methods:**

This is a retrospective analysis using IQVIA Longitudinal Patient Database. Starting from 18 + years old individuals with migraine, we selected two cohorts: subjects with triptans prescriptions before and no triptans prescriptions after Index Date (triptan withdraw) and subjects without triptans prescriptions both before and after Index Date (no triptan prescriptions). Index Date was the first record of a health encounter for migraine in 2019. Individuals with cardiovascular disease (CVD) within no triptan prescriptions group were also quantified.

**Results:**

Triptan withdraw and no triptan prescriptions cohorts numbered 605 and 3270, respectively, 5% and 29% of subjects with migraine. Mean age was 47 and 51 years respectively; women were more represented (~ 80%). Hypertension and thyroid disease were most frequent comorbidities; non-steroidal anti-inflammatory drugs were among most frequently recorded treatments. Subjects with CVD within no triptan prescriptions cohort were 621 and with triptan withdraw cohort subjects represented the basis to estimate those who might benefit from alternative options for the acute treatment of migraine, who were around 60,000 and accounted for 11% of subjects seeking primary care due to migraine.

**Conclusions:**

This analysis provides a real-word estimate of Italian people that might benefit from different therapeutic approaches as an alternative to triptans, which sometimes might be not effective and/or poorly tolerated. Such estimate should be intended as the lower limit of a wider range due to strict criteria adopted.

**Supplementary Information:**

The online version contains supplementary material available at 10.1007/s10072-024-07493-w.

## Introduction

Migraine is a common neurological disorder whose onset is usually in adolescence or early adult life [1] and presents with symptoms which affect people’s quality of life limiting their participation in work, family, and social activities [2]. Indeed, migraine has been classified by the 2019 Global Burden of Disease Study (GBDS) as the second overall (both genders, all ages) cause of years lived with disability (YLDs), but takes first place in young women [3]. Migraine can be classified based on headache frequency: Episodic migraine (EM) is characterized by < 15 headache days per month, whereas chronic migraine (CM) is characterized by ≥ 15 headache days per month [4]. In Italy, the prevalence of migraine was estimated [5] between 12 and 14% and determines, on average, a total direct cost per patient per year ranging between € 427 and € 829 for EM, and between €2037 and € 2648 for CM [5].

Once a correct diagnosis of migraine is made, pharmacotherapy can be either acute or preventive. Current acute migraine treatment include non-steroidal anti-inflammatory drugs (NSAIDs) and other analgesics and triptans [6]. Triptans have been specifically developed to treat migraine and represent the gold standard for acute treatment of migraine when first-line treatment with NSAIDs and other analgesics is not sufficiently effective [7]. Indeed, triptans are effective in treating acute migraine and are prescribed not only in headache centers and general neurology, but also in primary care [8]. However, some patients experience inadequate pain relief or headache recurrence; also, some patients might present contraindications for triptans. For these reasons, there is still a need for alternative options for the treatment of acute migraine [7].

The identification of individuals with unmet needs for acute migraine treatment was deemed to be a matter of great importance to optimize treatment in clinical practice by the European Headache Federation (EHF). Under this perspective, EHF recently published a consensus paper providing definitions of effective treatment of a migraine attack, triptan-responder, and triptan failure [8]. The definition of *effective treatment of a migraine attack* is not specific for triptans and requires reaching a well-being status within 2 h from intake of the drug. The well-being status should be maintained for at least 24 h and is determined by the occurrence of all the following: (1) improvement of headache from severe or moderate to mild or absent, (2) absent or minimal disturbances due to migraine-related non-pain symptoms, and (3) no meaningful drug-related adverse events [8]. A patient is defined as *triptan-responder* to a given triptan when the drug leads to effective attack treatment in at least 3 out of 4 consecutive attacks [8]. The concept of *triptan failure* accounts for cases where the condition of triptan-responder does not occur, and it translates in different definitions according to the number of triptans to which a patient cannot be considered a responder. In particular, a patient is defined as *triptan non-responder* in case of failure of a single triptan, *triptan-resistant* in case of failure of at least 2 triptans, and *triptan-refractory* in case of failure of at least 3 triptans [8]*.* Finally, the consensus paper defines *triptan ineligibility* as the presence of an acknowledged contraindication as reported in the summary of product characteristics (SPC) [8].

According to EHF, people who are triptan resistant or refractory are highly in need of alternative drug classes to respond to a persistent therapeutic unmet need [8]. The presence of a considerable unmet need for people with insufficient efficacy and/or tolerability to triptans was confirmed by a systematic literature review by Leroux and colleagues, irrespective of the definitions or methodologies applied to identify such population [9]. In addition, treatment options different from triptans are advisable in those individuals matching the definition of triptan ineligibility [8].

To authors’ knowledge, no attempts have been made until now to provide a real-world evidence-based estimate of the overall unmet need in the acute treatment of migraine in Italy. Being so, the present analysis used secondary data from a big sample of Italian general practitioners (GPs) to (1) describe subjects with migraine who interrupted treatment with triptans and those who did not receive prescriptions for triptans and (2) provide a national-level estimate of the number of people who might benefit from different therapeutic approaches as an alternative to triptans for acute migraine treatment.

## Material and methods

### Data source

The present analysis used data from the IQVIA Italian Longitudinal Patient Database (LPD). IQVIA LPD provides information from a representative sample of GPs who, according to Italian law requirements, use an ambulatory management software to record information on their patients’ routine visits. Recorded data include diagnoses, drug prescriptions, medical, and demographic data. The codification system of diagnoses follows the International Classification of Diseases 9th revision (ICD-9), while that of drugs complies with the Anatomical Therapeutic and Chemical (ATC) classification. GPs voluntarily agreed to contribute to the database and attended specific trainings for data entry. Currently, about 900 GPs contribute to the database, providing data of approximately 1.2 million patients, who are representative of the Italian general population managed by GPs in terms of age and gender [10]. Italian IQVIA LPD has been shown to be a reliable source of information in numerous previous studies and disease areas [11–14], including neurology [6, 15, 16].

### Populations and rules

**Fig. 1:**
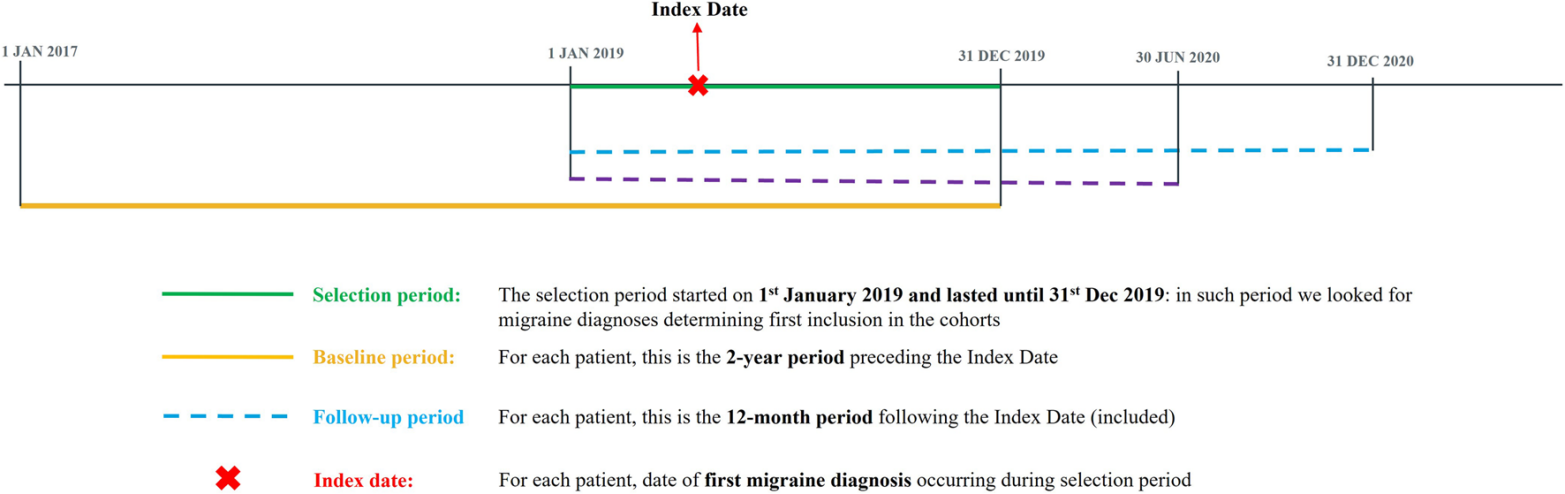
Definition of Index Date and period of interest (baseline and follow-up)

We first included subjects aged 18 years or older with at least one occurrence of a record of a health encounter related to a diagnosis of migraine (ICD-9 code 346) during 2019 (i.e., *selection period*). An *Index Date* was defined for each subject according to the date of registration of the first migraine record during the selection period. Records of health encounters related to migraine could preexist, as the Index Date should not necessarily coincide with the first diagnosis of migraine. Among the included individuals, we selected two mutually exclusive cohorts: the cohort of migraine subjects who had a triptan prescription but interrupted the treatment (triptan withdraw) and that of migraine subjects without prescriptions of triptans (no triptan prescriptions). To be included in the cohort of triptan withdraw, subjects had to have at least one prescription of at least one triptan during the 2-year period preceding the Index Date (i.e., *baseline period*), and no triptans prescriptions during the 12-month period starting at the Index Date (i.e., *follow-up period*). To be included in the cohort of no triptan prescriptions subjects, individuals must not have triptans prescriptions neither during the baseline period, nor during follow-up, but shall present with an additional record of a health encounter related to migraine that served as diagnosis confirmation during baseline and/or follow-up period. An additional subgroup was identified starting from the cohort of no triptan prescriptions by selecting all subjects who, during the baseline period, presented at least one registration of a cardiovascular (CV) diagnosis falling among triptans contraindications according to Dodick and colleagues [17] (please see Fig. 1 for an exemplification of the adopted design).

### Information extracted from the database

Information extracted from the database to characterize the cohorts included subjects’ age, sex, drugs’ prescriptions, neurologist visits referrals, and comorbidities recorded during baseline period. Comorbidities of interest were defined based on previous literature and included dyspepsia, irritable bowel syndrome, asthma, thyroid diseases, essential hypertension, anxiety, and depression [1, 6]. Finally, information specifically extracted for triptan withdraw cohort consisted of drugs specifically prescribed in relation to a diagnosis of migraine as per GP recording during follow-up; information specifically extracted for the cohort of no triptan prescriptions were recording of CV diagnoses identified by Dodick and colleagues [17] as contraindications to triptans use according to technical schedules during the baseline period (please see Supplementary Material for the list of ICD-9 codes defining comorbidities and CV contraindications).

### Statistical analysis

Descriptive statistics were used to provide an overview on demographic and clinical characteristics of both cohorts, and all the analyses were performed using SAS® Studio 3.8. Moreover, the sum of the number of subjects belonging to the (1) cohort of triptan withdraw and (2) subgroup of no triptan prescriptions cohort who also had a registration for a CV conditions represented the basis to provide the estimate of the number of Italian subjects who might benefit from drugs other than triptans for acute migraine treatment because they had to interrupt triptans or were not prescribed with triptans due to contraindications. Indeed, those individuals were sampled from around 1 million of active adult patients into IQVIA LPD in 2019, who, in turns, were representative of the Italian adult population in 2019, that, according to ISTAT, numbered around 50 millions of people [18]. Based on these figures, the sum of the number of subjects in triptan withdraw cohort and of those belonging to the subgroup of no triptan prescriptions subjects who also have CV contraindications was extrapolated to get the corresponding national-level estimate according to the below formula:

1 $$X :W=N :Z; X=\frac{N}{Z}\times W; X=\frac{N}{\mathrm{1,037,592}}\times \mathrm{50,243,518}$$

where *X* is the national-level estimate of subjects who might benefit from drugs other than triptans for acute migraine treatment, *W* the number of 18 + Italian residents in 2019 according to ISTAT, *N* the sum of the number of subjects in triptan withdraw cohort and of those belonging to the subgroup of no triptan prescriptions subjects who also have CV contraindications, and *Z* the number of 18 + active patients into IQVIA LPD in 2019.

The same approach was adopted to provide the national-level estimate of the number of Italian adult subjects who seek care from GPs due to migraine. The basis to provide such estimate was the number of adult people who had at least one occurrence of a record of a health encounter related to a diagnosis of migraine during 2019.

## Results

### Subjects’ identification

**Fig. 2:**
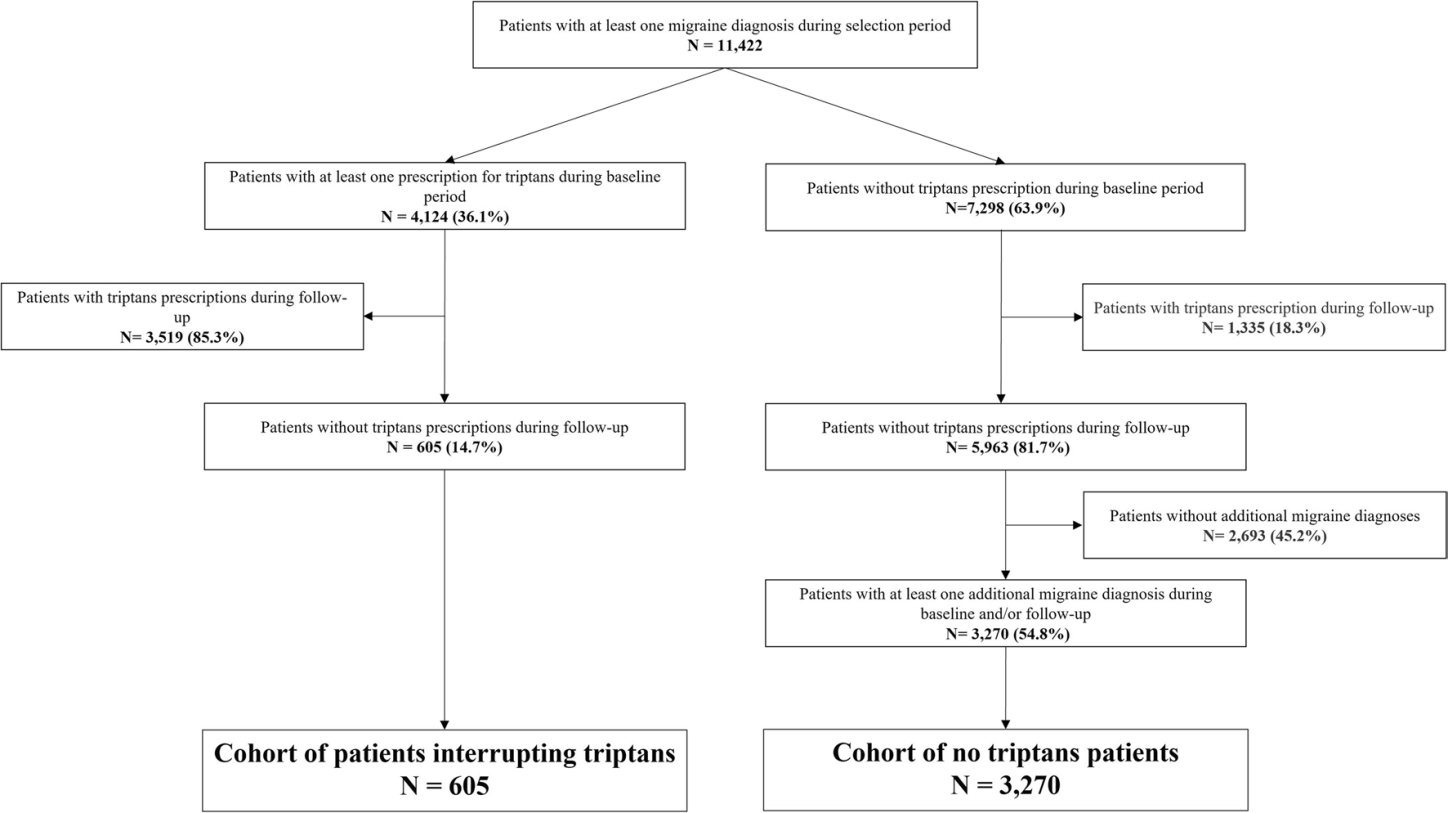
Flow diagram of patients interrupting triptans and no triptans patients

Among subjects with available data during the entire period of interest, those who were aged 18 years or older and had at least one record of a health encounter for migraine during the selection period were 11,422. There were 4124 (36.1%) individuals with prescriptions of at least one triptan during baseline period who, once having excluded subjects with triptans’ prescription during follow-up, led to the cohort of triptan withdraw, which was composed of 605 individuals; on the other hand, we found 7298 (63.9%) subjects without any triptans prescription during baseline: Once excluded subjects with triptans prescriptions during follow-up and those without a migraine diagnosis confirmation, we got the cohort of no triptan prescriptions subjects, which was composed of 3270 individuals (Fig. 2); of them, 621 (19.0%) had a recorded CV condition.

### Demographic and clinical characteristics

Subjects of both cohorts were predominantly women (79.7% and 77.4%, respectively); Mean age was 47 years for triptan withdraw cohort and 51 years for no triptan prescriptions cohort (Table 1). Among comorbidities of interest, essential hypertension and thyroid diseases were the most frequently reported conditions; hypertension was reported for almost 28% of triptan withdraw cohort, while thyroid diseases accounted for 21%. For both groups, proportions of subjects affected by asthma, depression, and anxiety ranged from 7 to 10%, while those with dyspepsia and/or irritable bowel syndrome did not reach 5%. Neurologist’s visit referrals during baseline were recorded for one-third of triptan withdraw cohort, while subjects with at least one request accounted for 20% of no triptan prescriptions cohort (Table 1).

**Table 1:** Demographic and baseline clinical characteristics of patients interrupting triptans and no triptans patients

Patients characteristics		Patients interrupting triptans (*N* = 605)		No triptans patients (*N* = 3270)	
Sex					
Female	*n* (%)	482	(79.7)	2,532	(77.4)
Age at Index Date					
Mean (SD)		46.7	(14.1)	51.3	(16.3)
Age class at Index Date					
< 40	*n* (%)	188	(31.1)	790	(24.2)
40–49	*n* (%)	150	(24.8)	709	(21.7)
50–59	*n* (%)	164	(27.1)	784	(24.0)
60–69	*n* (%)	70	(11.6)	504	(15.4)
70 +	*n* (%)	33	(5.5)	483	(14.8)
Comorbidities^a^					
Dyspepsia	*n* (%)	27	(4.5)	155	(4.7)
Irritable bowel syndrome	*n* (%)	26	(4.3)	156	(4.8)
Asthma	*n* (%)	56	(9.3)	253	(7.7)
Thyroid disease	*n* (%)	115	(19.0)	674	(20.6)
Essential hypertension	*n* (%)	131	(21.7)	911	(27.9)
Anxiety	*n* (%)	54	(8.9)	313	(9.6)
Depression	*n* (%)	45	(7.4)	314	(9.6)
Neurologist visit referrals					
Yes	*n* (%)	202	(33.4)	647	(19.8)

^a^Numbers and proportions of patients with at least one recording of the corresponding diagnosis. One patient can be counted in more than one group

### Co-treatments

**Fig. 3:**
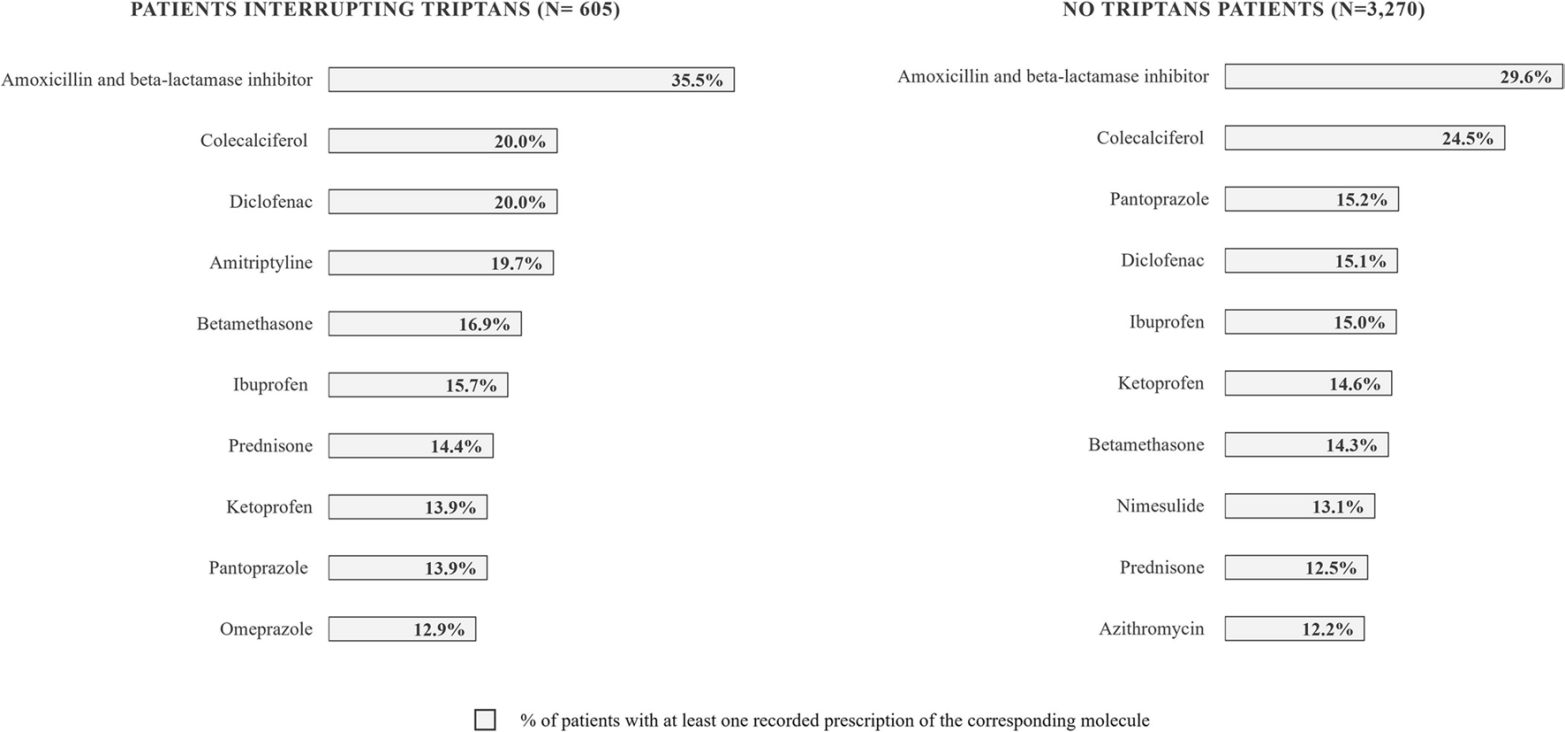
Top 10 of most frequently prescribed molecules during baseline period for patients interrupting triptans and no triptans patients

The most frequently prescribed molecules during baseline period were amoxicillin and beta-lactamase inhibitor (recorded for 35.5% and 29.6% of triptan withdraw and of no triptan prescriptions cohort, respectively) and colecalciferol (recorded for 20.0% and 24.5% of triptan withdraw and of no triptans prescriptions cohort, respectively). Of note, among top 10 most frequently prescribed treatments, we found 3 non-steroidal anti-inflammatory drugs (NSAIDs) molecules for treatment withdraw cohort and 4 NSAIDs molecules for no triptan prescriptions one (Fig. 3). The analysis on treatments specifically prescribed for migraine during follow-up for the cohort of triptan withdraw revealed that the most frequently recorded molecule was amitriptyline, which was found for around 10% of the patients, followed by topiramate (6.8%); among the most frequently prescribed drugs, we also found 5 molecules falling into NSAIDs class: ketoprofen (4.3%), ketorolac (4.3%), nimesulide (4.0%), ibuprofen (3.6%), and indometacin (3.3%); prescriptions of fixed-dose combinations including paracetamol were found for 3.5% of the subjects (data not shown).

### CV contraindication

Within the cohort of no triptan prescriptions subjects, 621 (19.0%) had a recording of a CV diagnosis during baseline period (Table 2). In particular, cerebrovascular disease, uncontrolled hypertension, and other significant underlying CV diseases were all reported for more than 3% of the subjects in the cohort (Table 2).

**Table 2:** Presence of cardiovascular conditions representing triptans contraindications among no triptans patients

Patients characteristics		No triptans patients (*N* = 3270)	
Presence of CV conditions			
Yes	*n* (%)	621	(19.0)
CV condition^a^			
Ischemic heart disease	*n* (%)	73	(2.2)
Cerebrovascular disease	*n* (%)	165	(5.1)
Peripheral artery disease	*n* (%)	68	(2.1)
Uncontrolled hypertension	*n* (%)	113	(3.5)
Gastrointestinal ischemia	*n* (%)	< 5	
Other Significant Underlying CV Disease	*n* (%)	358	(11.0)

^a^Numbers and proportions of patients with at least one recording of the corresponding diagnosis/molecule. One patient can be counted in more than one group

### Estimate of subjects who might benefit from different therapeutic approaches as an alternative to triptans for acute migraine treatment

Subjects with migraine who interrupted treatment with triptans (i.e., triptan withdraw cohort) were 605 on IQVIA LPD: This number translated into an estimate of around 29,300 individuals who interrupt treatment with triptans over a 1-year period and at national level; within the cohort of no triptan prescriptions, subjects with a recorded CV condition were 621 based on IQVIA LPD data: This number translated into an estimate of around 30,100 patients who have CV contraindications over a 1-year period and at national level. The sum of the above estimates led to an overall one of around 60,000 Italian subjects with migraine who might benefit from different therapeutic approaches as an alternative to triptans for acute migraine treatment to respond to a persistent unmet need. Adult subjects who had at least one health encounter with the GP due to migraine during 2019 were 11,422 and translated into an estimate of around 553,000 people. Being so, Italian subjects with migraine who might benefit from different therapeutic approaches as an alternative to triptans for acute migraine treatment represented around 11% of individuals seeking for primary care due to migraine.

## Discussion

This analysis used Italian GPs data to (1) describe subjects with migraine who interrupted treatment with triptans and those who did not receive prescriptions for triptans and (2) provide a national-level estimate of the number of people who might benefit from different therapeutic approaches as an alternative to triptans for acute migraine treatment.

Six hundred five adult subjects out of 4124 (15%) identified as being affected by migraine during 2019, and previously receiving triptans were found to not have further prescriptions of triptans during the subsequent year. Past evidence reported that about 30% of individuals do not respond adequately to triptans because of poor efficacy or tolerability [19], and it has been reported that a considerable subgroup of triptan users is dissatisfied with their care and most of them would be willing to try alternative acute medications [20]. Also, real-world studies have shown triptans’ low persistency and low rates of subsequent prescriptions following treatment initiation in the United States and Europe [21]. A study by Pavone and colleagues used the drug prescription database of a regional Health Authority in Italy to investigate on patterns of triptans utilization: Among subjects receiving at least one triptans prescription, those with a single prescription accounted for 60% [22]. The proportion of individuals interrupting treatment with triptans observed in the present analysis might be seen as a proxy of triptans’ suboptimal efficacy and/or tolerability.

Findings from this analysis showed that subjects who did not receive triptans prescriptions by GPs during the entire period of interest were 3270; they accounted for 29% of adults who had a diagnosis of migraine and certainly include those who have contraindications to triptans. Prior evidence suggested that the proportion of subjects with migraine who are contraindicated to triptans due to CV conditions is around 20% [17, 23].

According to findings from the present analysis, the number of Italian patients who might benefit from different therapeutic approaches as an alternative to triptans for the treatment of acute migraine was conservatively estimated to be around 60,000, and they accounted for 11% of adult subjects seeking care from GPs due to migraine. The authors believe that such estimate should be intended as the lower limit of a wider range mainly for two reasons. First, due to the inclusion criterion imposing total absence of triptan prescriptions during follow-up to identify subjects experiencing triptans’ poor efficacy and/or tolerability, and in light of previous findings quantifying inadequate response to triptans [19], the extent of triptans’ poor efficacy and/or tolerability here observed might be underestimated. Indeed, it cannot be excluded that, in absence of easily accessible migraine-specific alternative treatment options, some patients still assume triptans despite unsatisfactory effectiveness or in case of mild adverse events occurrence. Second, because prior evidence suggested that the proportion of subjects with migraine presenting triptans contraindications is around 20% [17, 23], and because some individuals might successfully abort migraine attacks without assuming triptans (i.e., assuming non migraine-specific drugs), our national-level estimate of subjects with migraine and triptans contraindications solely relied on subjects actually presenting CV conditions. Such subgroup accounted for 19% of patients without triptans prescriptions, and for 5% only of subjects with migraine.

The characterization of people affected by migraine included in the present analysis is coherent with previous findings. The vast majority of subjects in both cohorts were female, and the mean age was 47 years for the cohort of triptan withdraw and 51 years for the cohort of no triptan prescriptions. Gender differences in migraine epidemiology are well documented, and much higher proportions of women among subjects affected by migraine have been reported [6, 24–27]. Furthermore, previous studies have indicated that migraine peaks in middle life, and its frequency and severity decrease with age [1, 28]. In terms of clinical features, hypertension was the most common comorbidity, followed by thyroid diseases. A positive association between migraine and hypertension has been previously reported [29] and a high prevalence of hypertension among people suffering from migraine has been observed in different studies [1, 24]. Furthermore, a recent extensive literature research concluded that epidemiological studies suggest a relationship between migraine and thyroid dysfunction, even if the nature of the relationship remains unclear [30].

The distribution of most frequently prescribed molecules during baseline for both cohorts was in line with data from the 2019 Report by The Medicines Utilization Monitoring Centre (OSMED) published by the Italian Medicines Agency (AIFA) [31]. Among molecules most frequently prescribed to treat migraine following triptans interruption, we found NSAIDs and analgesics: Despite there might be some patients for whom NSAIDs and/or analgesics are effective in treating migraine episodes, evidence showed that subjects turning to traditional acute treatment for migraine might experience increasing disability and/or increasing migraine frequency [23, 32]. Within triptan withdraw cohort, one-third had referrals for neurological visits, with this being in line with findings from *My Migraine Voice* survey that reported that 32% of migraine subjects visited a neurologist during the 6-month period preceding the interview [5]. Within no triptan prescriptions cohort, those with neurologist visits referrals accounted for 20% only. According to the authors’ opinion, on the one hand, this finding might be suggestive of a sort of disenchantment of subjects with triptans contraindication who, not being aware of alternative options or because getting used to live with migraine, do not seek care from specialists. On the other hand, it is also possible that drugs that are not specific to treat acute migraine are somewhat effective; thus, patients do not need to see a specialist.

Among main strengths of the present analysis, there is the very large and general-population representative database [10]. Indeed, subjects’ description here reported has shown to be comparable with findings from previous studies conducted on people affected by the same condition [1, 6, 24–28]. Furthermore, the adoption of GPs’ perspective allowed avoiding the selection bias that might affect studies which relies on patients managed in the specialistic context and might thus offer a partial overview on migraine. On the other hand, results from the current analysis should also be interpreted in the context of some limitations related to its retrospective and descriptive nature. First, identification of cohorts relied on proxies rather than on ad hoc collected information as typical for outcome research studies conducted using secondary data. Second, only data on written prescriptions were available; therefore, we assumed that any written prescription was actually consumed. However, rates and volumes of prescriptions by GPs, such as those obtained from the IQVIA Italian LPD, have been already shown to be consistent with those measured by data sources providing information on dispensed medications [33]. Third, the absence of triptans prescriptions during follow-up required for inclusion in the cohort of triptan withdraw might reflect an improvement of migraine condition determined by ageing. However, age distribution of subjects included in the cohort makes authors confident that such bias, even if present, would only partially affect findings and would be counterbalanced by the conservative approach adopted. Fourth, the cohort of no triptan prescriptions might include both individuals who assume drugs which are not migraine-specific but effective in treating acute episodes, as well as individuals who receive a proper and successful migraine prophylaxis: Both aspects were not specifically investigated due to limitations relying on the data source which cannot provide an exhaustive picture due to the under-reporting of drugs not requiring a clinician’s prescription and the absence of information on prophylaxis drugs falling outside primary care context. For this reason, authors preferred to provide a conservative estimate of the number of Italian subjects with contraindications to triptans by including only those with a documented CV diagnosis. Fifth, it was not possible to retrieve information on the number of migraine attacks experienced by subjects included in the analysis as this information is not directly recorded by GPs. Finally, we were not able to distinguish between individuals interrupting triptans because of poor efficacy or tolerability; thus, we provided an overall estimate of subjects who interrupted triptans no matter the underneath reason.

## Conclusion

Triptans are specifically indicated to treat migraine and prescribed not only in headache centers and general neurology, but also in primary care. However, it is known that there are subjects for which triptans are not effective and/or well tolerated, or who present with contraindications to their use. Under this perspective, the present analysis provides a useful real-word based estimate of the number of Italian people that might potentially benefit from alternative pharmacology options. Indeed, there is still an important therapeutic unmet need for these patients, and its resolution might lead to an improvement in terms of quality of life.

### Supplementary Information

Below is the link to the electronic supplementary material.

Supplementary file1 (DOCX 22 KB)

## Data Availability

The data that support the findings of this work are available from IQVIA, but restrictions apply to the availability of these data, which were used under license for the current work, and so are not publicly available. Data are however available from the corresponding author upon reasonable request and with permission of IQVIA.
