# Supplementary material for: Acute treatment of migraine: quantifying the unmet need through real-world data in Italy
Source: Neurol Sci. 2024 Mar 27;45(9):4427–35. doi: 10.1007/s10072-024-07493-w (PMC11306385; doi:10.1007/s10072-024-07493-w)
Supplement: Supplementary file 1 — Supplementary file1 (DOCX 22 KB) [file 10072_2024_7493_MOESM1_ESM.docx]

**Supplementary Materials**

**Acute treatment of migraine: quantifying the unmet need through real-world data in Italy**

Simona Sacco^1^, Sonia Di Ciaccio^2^, Roberto Di Virgilio^3^, Valeria Pegoraro^4*^, Raffaele Ornello^1^

^1^ Department of Biotechnological and Applied Clinical Sciences, University of L'Aquila, L'Aquila, Italy

^2^ Pfizer Srl, Medical Department, Rome, Italy

^3^ Pfizer Srl, Health Economics and Outcome Research Department, Rome, Italy

^4^ RWS Department, IQVIA Solutions Italy S.r.l, Milan, Italy

Valeria Pegoraro, MSc *

IQVIA Solutions Italy S.r.l.

Address: Via Fabio Filzi 29, 20124. Milan, Italy

Tel: +39 3339326866

e-mail: [valeria.pegoraro@iqvia.com](mailto:valeria.pegoraro@iqvia.com)

ORCID-ID: 0000-0003-2359-6380

*Table 1S. International Classification of Diseases 9^th^ revision* (*ICD-9) codes used to determine comorbidities of interest*

| **Condition** | **ICD-9 Code** |
| --- | --- |
| Dyspepsia | 536.8x |
| Irritable bowel syndrome | 564.1x |
| Asthma | 493.xx |
| Thyroid disease | 240.xx, 241.xx, 242.xx, 243.xx, 244.xx, 245.xx, 246.xx |
| Essential hypertension | 401.xx |
| Anxiety | 300.0x |
| Depression | 296.2x, 296.3x, 296.5x, 296.6x, 296.82, 300.4x, 309.0x, 309.1x, 309.28 |

*Table 2S. International Classification of Diseases 9^th^ revision* (*ICD-9) codes used to determine tension headache*

| **Condition** | **ICD-9 Code** |
| --- | --- |
| Tension headache | 307.81 |

*Table 3S. International Classification of Diseases 9^th^ revision* (*ICD-9) codes used to determine cardiovascular (CV) contraindications to triptans*

| **Condition** | | **ICD-9 Code** |
| --- | --- | --- |
| **Ischemic heart disease** | | 410.xx, 411.xx, 412.xx, 413.xx, 414.xx |
| **Cerebrovascular disease** | | 430.xx, 431.xx, 432.xx, 433.xx, 434.xx, 435.xx, 436.xx, 437.xx, 438.xx |
| **Peripheral artery disease** | | 440.xx, 443.0x, 785.4x, 443.1x, 443.8x, 443.9x, 249.70, 250.70, 250.71, V43.4x, V45.89 |
| **Uncontrolled hypertension** | | 402.xx, 403.xx, 404.xx, 405.xx, 437.2x, 362.11 |
| **Gastrointestinal ischemia** | | 557.0x |
| **Other significant underlying CV disease** | |  |
|  | Cardiac surgery and/or implants | V43.3x, V42.2x, V45.02, V43.21, V43.22, V45.09, V43.4x, V45.00 |
|  | Structural heart disease | 394.xx, 395.xx, 396.xx, 397.0x, 397.9x, 398.0x, 393.xx, 398.91, 397.1x, 398.90, 398.99, 424.xx, 421.1x, 425.11, 425.18, 428.xx, 745.xx, 746.xx, 747.0x, 747.10, 747.22, 747.31, 747.39, 747.29, 747.4x, 747.61 |
|  | Arrhythmia | 426.12, 426.13, 426.0x, 426.3x, 426.54, 426.82, 426.89, 427.1x, 427.0x, 427.2x, 427.3x, 427.4x, 427.8x, 427.9x |
|  | Other cardiac conditions | 427.5x, 441.xx, 444.0x, 444.1x, 444.2x, 444.8x, 444.9x, 780.2x, 794.3x |
